# Adaptive neural information processing with dynamical electrical synapses

**DOI:** 10.3389/fncom.2013.00036

**Published:** 2013-04-16

**Authors:** Lei Xiao, Dan-ke Zhang, Yuan-qing Li, Pei-ji Liang, Si Wu

**Affiliations:** ^1^Department of Biomedical Engineering, Shanghai Jiao Tong UniversityShanghai, China; ^2^School of Automation Science and Engineering, South China University of TechnologyGuangzhou, China; ^3^State Key Laboratory of Cognitive Neuroscience and Learning, Beijing Normal UniversityBeijing, China

**Keywords:** electrical synapses, short-term plasticity, information processing, adaptation, dynamical encoding

## Abstract

The present study investigates a potential computational role of dynamical electrical synapses in neural information process. Compared with chemical synapses, electrical synapses are more efficient in modulating the concerted activity of neurons. Based on the experimental data, we propose a phenomenological model for short-term facilitation of electrical synapses. The model satisfactorily reproduces the phenomenon that the neuronal correlation increases although the neuronal firing rates attenuate during the luminance adaptation. We explore how the stimulus information is encoded in parallel by firing rates and correlated activity of neurons, and find that dynamical electrical synapses mediate a transition from the firing rate code to the correlation one during the luminance adaptation. The latter encodes the stimulus information by using the concerted, but lower neuronal firing rate, and hence is economically more efficient.

## Introduction

In the central nervous system, neurons communicate with each other via two basic forms of synapse: chemical and electrical synapses (Kandel et al., 2000). A chemical synapse is asymmetric in structure, which passes information from a presynaptic neuron to a postsynaptic one through neurotransmitters release, and this occurs when the presynaptic neuron fires an action potential. An electrical synapse, on the other hand, is bidirectional, which allows signal to be transmitted in both ways. Compared to a chemical one, an electrical synapse is usually fast and underlies rapid communication among neighboring neurons of the same type.

It is well known that the strength of a chemical synapse can undergo a variety of short and long-term plasticity (Tsodyks and Markram, 1996; Bi and Poo, 1998; Dan and Poo, 2006). It has also been shown in experimental studies that the strength of an electrical synapse can be modulated similarly as a chemical one. For instances, it was found that titanic stimulation can lead to either long- or short-term potentiation of electrical synapses in goldfish (Yang et al., 1990; Pereda and Faber, 1996); in the rat thalamic reticular nucleus, titanic stimulation can cause long-term depression in the electrical synapses (Landisman and Connors, 2005; Haas et al., 2011); and in the vertebrate retina, electrical synapses can be dynamically regulated by either ambient illumination or circadian rhythms (Bloomfield and Volgyi, 2009).

Although a large volume of experimental data has revealed the abundant existence and the plasticity of electrical synapses in the neural system, their functional roles in neural information processing remain largely unclear (Connors and Long, 2004). In the thalamic reticular nucleus, electrical synapses may contribute to the shift between arousal states (Haas et al., 2011). In the retina, electrical synapses are sensitive to the background light conditions (Bloomfield and Volgyi, 2009), and the synchronous activity of electrically coupled ON direction-selective ganglion cells may encode the direction information of a moving stimulus (Ackert et al., 2006). It was also found that retinal ganglion cells (RGCs) coupled with electrical synapses exhibit stronger concerted activity than connected (indirectly) with chemical synapses in a circuit (Brivanlou et al., 1998; Jing et al., 2010).

In the present study, we investigate a potential role of electrical synapses in processing stimulus information during luminance adaptation. We first explore the effects of electrical and chemical synapses on generating neural correlation. We find that the neuronal correlation strength is much more sensitive to the plasticity of an electrical synapse than to the plasticity of a chemical one, indicating the potential importance of electrical synapses in modulating synchrony of neuronal activities. We then propose a phenomenological model for short-term facilitation of electrical synapses, based on the experimental finding that during the luminance adaptation, the neuronal correlation strength increases whereas the firing rates attenuate. The proposed model satisfactorily reproduces the experimental data. Finally, we explore the computational role of dynamical electrical synapses, and find that they contribute to generate a transition in encoding properties during the adaptation. The implication of this transition is discussed.

## Materials and methods

### The neuron-pair models

**Figure 1:**
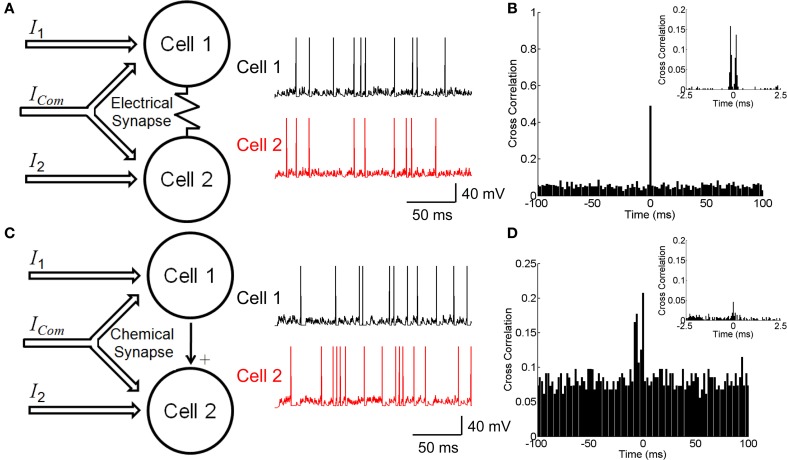
**Neuronal circuitry with electrical and chemical synapses. (A)** A model of electrically coupled neurons (left panel) and examples of simulated firing activities (right panel). Neurons receive external currents with both common and independent components. **(B)** The cross correlation function between electrically coupled neurons with bin size of 2 ms. Inset shows the cross correlation function with bin size of 0.1 ms. **(C)** A model of chemically connected neurons (left panel) and examples of simulated firing activities (right panel). Neurons receive external currents with both common and independent components. **(D)** The cross correlation function between chemically connected neurons with bin size of 2 ms. Inset shows the cross correlation function with bin size of 0.1 ms. The parameters values for the two conditions have been chosen to fit the two models to have similar behavior, which are: *C* = 0.5 nF, *g*_*L*_ = 0.025 μS, τ^*s*^ = 5 ms, μ = 0.62 nA, σ = 0.5 nA, *g*^es^ = 0.025 μS, *u* = 0.05 μS, *c* = 0 for the electrical synapse, *c* = 0.5 for the chemical synapse, and simulation step size = 0.1 ms.

To investigate the effects of electrical and chemical synapses on generating correlated neuronal responses, we construct neuron-pair models coupled by either an electrical or a chemical synapse as shown in Figures 1A,C.

For neurons coupled with an electrical synapse, the dynamics of neurons are written as

(1) $$\begin{array}{l} C\frac{dV_{i}(t)}{dt}=-g_{L}[V_{i}(t)-V^{\text{rest}}]+g^{\text{es}}[V_{j}(t-d^{\text{es}})\\ \;-V_{i}(t)]+I_{i}^{\text{ext}}(t),\;i,j=1,2 \end{array}$$

where *V*_*i*_ is the membrane potential of the *i*th neuron, *C* the membrane capacitance, *g*_*L*_ the leaky conductance, *V*^rest^ = −70 mV the resting potential, and *I*^ext^_i_ the external input current. *g*^es^ represents the conductance of the electrical synapse, which is a constant unless the synapse is undergoing plasticity. *d*^es^ denotes the transmission delay of the electrical synapse, which is in the range of 0.2–0.4 ms according to the experimental data (Brivanlou et al., 1998; Li et al., 2012). The neuron fires when its membrane potential reaches to a threshold *V*^th^ = −50 mV, and *V*_*i*_ is reset to be *V*^reset^ = −70 mV after firing.

For neurons connected by a chemical synapse, the dynamics of neurons are written as

(2) $$\begin{array}{l} C\frac{dV_{i}(t)}{dt}=-g_{L}[V_{i}(t)-V^{\text{rest}}]-g_{ij}^{\text{cs}}(t-d^{\text{cs}})[V_{i}(t)\\ \;-V^{\text{rev}}]+I_{i}^{\text{ext}}(i),\;i,j=1,2 \end{array}$$

where *V*^rev^ = 0 mV denotes the reversal potential. *g*^cs^_ij_ is the conductance of the chemical synapse from the neuron *j* to *i*, whose dynamics is given by

(3) $$\tau^{s}\frac{dg_{ij}^{\text{cs}}}{dt}=-g_{ij}^{\text{cs}}+u\sum_{m}\delta (t-t_{j}^{m})$$

where τ^*s*^ is the synaptic time constant, *t*^*m*^_*j*_ the moment when the *m*th spike of the *j*th neuron is generated, and *u* the increment of the chemical conductance due to a spike generation. *d*^cs^ denotes the transmission delay of the chemical synapse, which is in the range of 2–3 ms.

The external inputs to the neurons are given by (see Figures 1A,C)

(4) $$I_{i}^{\text{ext}}=\mu (t)+\sigma [\sqrt{1-c}\xi_{i}(t)+\sqrt{c}\xi_{c}(t)],\;i=1,2$$

where μ(*t*) is the mean of the inputs. ξ_*i*_(*t*) is Gaussian white noise of zero mean and unit variance. Noise processes of the two neurons are independent to each other, i.e., < ξ_*i*_(*t*) ξ_*j*_(*t*′) > = δ_*ij*_δ(*t* − *t*′). ξ_*c*_(*t*) denotes the common noise to both neurons. σ is the noise strength. The parameter 0 ≤ *c* ≤ 1 determines the correlation strength between the inputs to the two neurons.

### Measuring the correlation strength

To quantify the characteristics of neural response, we divided time into small bins. A spike train is symbolized into “0” and “1” within a time bin, where *r*_*i*_ (*t*) = 1 means that the cell *i* fires in the *t*th time bin and “0” means that it does not fire. We use cross-correlation function (*CCF*) to measure the correlation strength between neurons. The value of *CCF* between two spike trains is calculated to be

(5) $$CCF(\Delta t)=\frac{N\sum_{t\;=\;1+|\Delta t|}^{N-\left|\Delta t\right|}r_{1}(t)r_{2}(t+\Delta t)}{\left(N-2|\Delta t|\right)\sqrt{\sum_{t\;=\;1}^{N}r_{1}{\left(t\right)}^{2}\sum_{t\;=\;1}^{N}r_{2}{\left(t\right)}^{2}}}$$

where *r*_*i*_(*t*) = 0, 1, for *i* = 1, 2, denotes the spike train generated by the *i*th neuron at the moment *t* and *N* indicates the length of spike train. The peak value around zero lag of *CCF* is used to represent the neuronal correlation strength.

### The experimental data

In this study, we use two sets of experimental data. Both experiments were performed on isolated bullfrog retinas, and the experimental procedures and equipments have been described in detail in (Li et al., 2012; Xiao et al., 2012). The previous works did not study the model and the functional role of electrical synapses presented in this paper.

In the first experiment (Li et al., 2012), the bullfrog retina was exposed to flickering pseudo-random checker-boards for 100 s (frame refresh rate = 20 Hz), and a multi-electrode system was used to record the responses of RGCs simultaneously. Figures 4A,B present the experimental results. We use this set of data to fit the phenomenological model for short-term facilitation of electrical synapses during the luminance adaptation. The model is then applied to interpret the neural data in the second experiment.

In the second experiment (Xiao et al., 2012), the bullfrog retina was exposed to flicking pseudo-random checker-boards for 15 s followed by a sustained dark stimulation. The whole adaptation process to the dark stimulus lasted for about 5 s. Figures 5A,B present the experimental results. We use this set of data to explore the potential functional role of dynamical electrical synapses.

Both experiments were strictly conformed to the humane treatment and use of animals as prescribed by the Association for Research in Vision and Ophthalmology, and were approved by the Ethic Committee, School of Biomedical Engineering, Shanghai Jiao Tong University.

### Measuring the stimulus information carried by firing rate and correlation

Denote *p*(**r**|*s*) the conditional probability of observing the neural response **r** given the stimulus *s*. We regard the dark stimulation and the random flicking check-boards as two stimuli, which occur with equal probability, i.e., *p*(*s*) = 1/2. For two neurons, **r** = {*r*_1_, *r*_2_}. The bin size is 5 ms, unless it is stated specifically. The total amount of the stimulus information that can be extracted from the neuronal data is given by the mutual information (Shannon, 1948),

(6) $$I=-\int drp(r)\log_{2}p(r)+\int dr\sum_{s}p(s)p(r|s)\log_{2}p(r|s)$$

To decompose the stimulus information into portions carried by different features of neuronal activities, we choose to use the information measure I^*^, which is known to be directly linked to the decoding error of maximum likelihood inference based on a mismatched model (Wu et al., 2001; Oizumi et al., 2010). I^*^ quantifies the information gain when a mismatched neural encoding model *q*(**r**|*s*) is applied, and is calculated as (Merhav, 1994),

(7) $$I^{*}(q)={\text{max}}_{\beta }\tilde{I}(q,\beta )$$

(8) $$\tilde{I}\left(q,\;\beta \right)=-\int drp\left(r\right)\log_{2}\sum_{s}p\left(s\right)q{\left(r|s\right)}^{\beta }+\int dr\sum_{s}p\left(s\right)p\left(r|s\right)\log_{2}q{\left(r|s\right)}^{\beta }$$

where β is a parameter to be optimized. This information measure has been applied recently for studying neural coding (Oizumi et al., 2010). By choosing the form of *q*(**r**|*s*) properly, the amount of the stimulus information contained in different features of neural responses can be obtained.

When two spike trains (binary variables) are considered, the joint probability of neural responses can be written as (Amari, 2001),

(9) $$p(r|s)=\frac{1}{Z}\exp\text{​}\left(\sum_{i}\theta_{i}^{1}r_{i}+\sum_{i\;<\;j}\theta_{ij}^{2}r_{i}r_{j}\right)$$

where *Z* is the normalization factor, and the parameters θ^1^_*i*_ is related to the firing rate of the *i*th neuron, θ^2^_*ij*_ is related to the correlation between the *i*th and *j*th neurons. The values of θ^1^ and θ^2^ can be uniquely determined by matching *p*(**r**|*s*) with the real distribution of the data.

Suppose we choose *q*(**r**|*s*) to be the probability distribution which has the same firing rates as *p*(**r**|*s*) but with vanishing correlation between neurons, i.e.,

(10) $$q(r|s)=\frac{1}{Z_{1}}\exp\text{​}\left(\sum_{i}\theta_{i}^{1}r_{i}\right)$$

where *Z*_1_ is the normalization factor. The parameter θ^1^_*i*_ is determined by the requirement that the firing rates remain the same for both distributions *p* and *q*. Thus, the value I^*^(*q*), refer to as *I*_1_ hereafter, is the amount of the stimulus information contained in the firing rates of neurons. Its discrepancy to the mutual information, denoted as *I*_2_ = *I* − *I*_1_ hereafter, is the amount of the stimulus information contained in the correlation. The relative contributions of firing rate and correlation are measured by the ratios, *R*_*i*_ = *I*_*i*_/*I*, for *i* = 1, 2.

## Results

### Neural correlations generated by electrical and chemical synapses

Neurons can be connected by either electrical or chemical synapses. We investigate how different forms of synapse affect neuronal correlation. The neuron-pair models coupled by either an electrical or a chemical synapse as shown in Figures 1A,C are used (see Materials and Methods). The correlation strength is measured by the *CCF* between the spike trains generated by two neurons.

Figure 1B shows the *CCF* for the electrically coupled neurons. We see that the *CCF* exhibits a narrow peak for the bin size of 2 ms, indicating that the two neurons' responses are largely synchronized. If the bin size is 0.1 ms, the *CCF* has dual peaks around Δ*t* = 0 due to the transmission delay of the electrical synapse (Figure 1B inset). These results agree with the experimental data for electrically coupled RGCs in the bullfrog retina (Li et al., 2012).

Figure 1D shows the *CCF* for the neurons connected via a chemical synapse. We see that the *CCF* has a much broader distribution than for the electrical synapse. This property is general and reflects that a chemical synapse is slow and that the correlation it generates is usually small.

**Figure 2:**
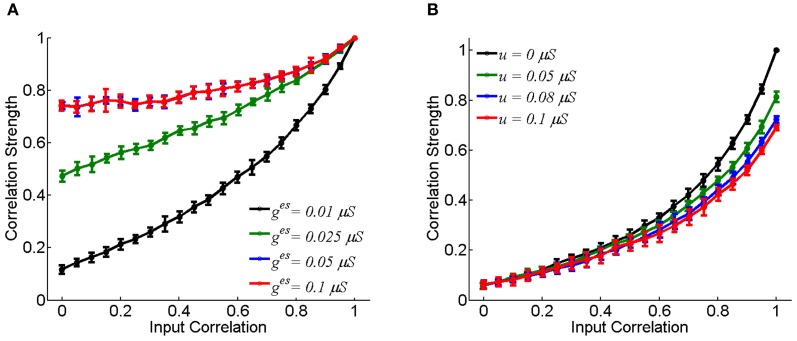
**The effect of coupling strength on the correlation strength between neurons.** In the simulation, 10-s data is generated and repeated for 10 times. Bin size is 2 ms, and error bars indicate Mean ± s.e.m. **(A)** For the electrical synapse. **(B)** For the chemical synapse. The parameters are the same as in Figure 1, but varying parameter *c*.

Figure 2 displays how the synaptic strength affects the neuronal correlation strength. For the electrical synapse, the correlation strength varies significantly for different conductance values of *g*^es^ (Figure 2A). On the other hand, for the chemical synapse, the correlation strength is rather insensitive to the coupling parameter *u* (Figure 2B, the chemical conductance *g*^cs^ increases with *u*). In the case of electrical synapse, the neuronal correlation can be very strong even when the input correlation is very small (for very small *c-values)*; whereas, in the case of chemical synapse, the neuronal correlation can only be strong when the input correlation is sufficiently large (for very large *c*-values). An intuitive justification for this is that a chemical synapse is slow and its effect on coordinating neuronal activities is diminished by input noises and the resetting of the membrane potential after neural firing.

We have only presented the result for the case that there is a single excitatory chemical synapse from the neuron 1 to 2 (see Figure 1B). For the case that there exists a reciprocal chemical synapse from the neuron 2 to 1, the property about correlation strength shown in Figure 2B still holds (data not shown).

In the present study, the membrane potential of a neuron after firing was reset to be the resting value, i.e., *V*^reset^ = V^rest^ = −70 mV. Alternatively, we could reset the neuron to be hyperpolarized after firing, e.g., *V*^reset^ = −85 mV. We found that this did not change our results qualitatively, and that hyper-polarization tended to increase the robustness of neural correlation mediated by gap-junction to noises.

**Figure 3:**
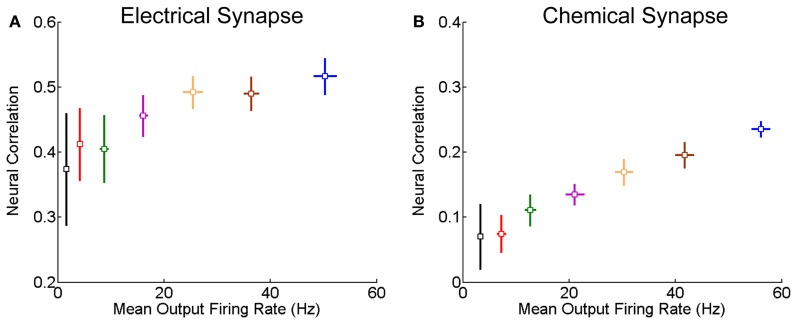
**Correlation strength vs. firing rate of neurons.** The mean of the inputs μ increases from 0.5 to 0.62 nA, and the step is 0.02 nA, and σ = 0.5 nA. Other parameters are the same as in Figure 1. In the simulation, 10-s data is generated and repeated 10 times for each condition. Error bars indicate Mean ± s.e.m. **(A)** For the neuron pair coupled by the electrical synapse; **(B)** For the neuron pair connected by the chemical synapse.

We further check for fixed synaptic strength, how the correlation strength changes with the neuronal firing rates. As expected, the correlation strength increases with the firing rates (Figure 3). This is understandable, since larger firing rates enlarge the effects of neuronal interaction via both forms of synapse.

### A phenomenological model for short-term facilitation of electrical synapse

We explore how an electrical synapse may vary with time during the adaptation of neuronal responses. The experiment was performed on an isolated bullfrog retina, which was exposed to flickering pseudo-random checker-boards for 100 s (Li et al., 2012; see Materials and Methods). A multi-electrode system was used to record the responses of RGCs simultaneously.

**Figure 4:**
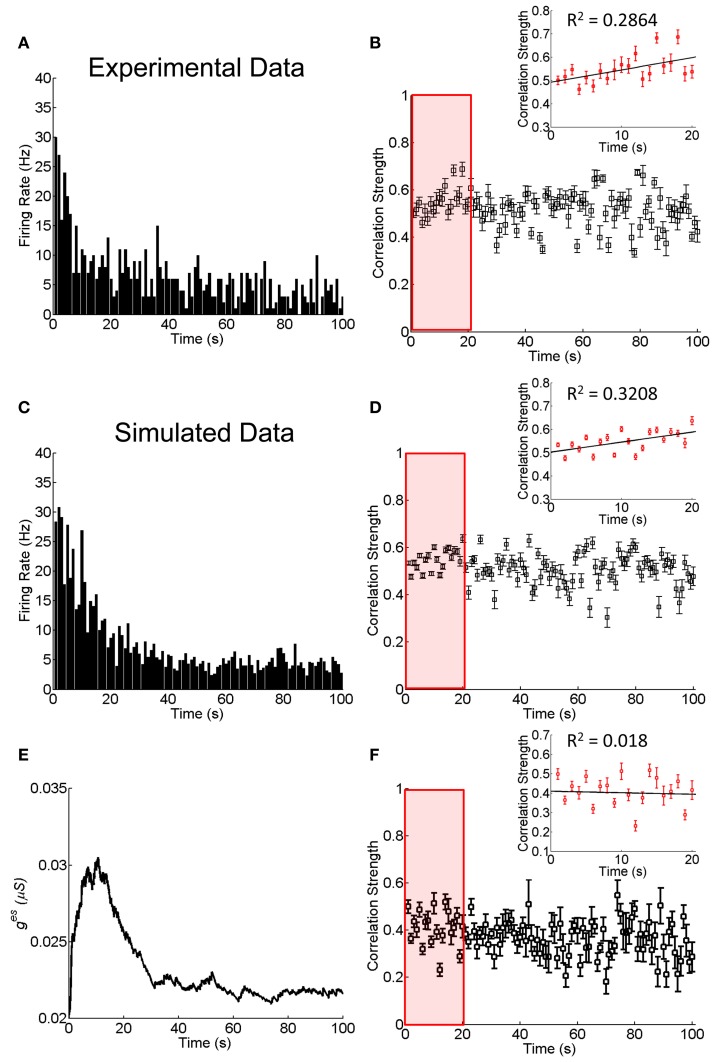
**Experimental and simulated results about the change of the correlation strength between two electrically coupled neurons during the adaptation to 100-s flicking pseudo-random checker-boards. (A)** An example of retinal ganglion cell's response to 100-s flicking pseudo-random checker-boards. The time window for calculating firing rate is 1 s. **(B)** The change of the correlation strength measured in the experiment. Inset highlights the change of the correlation strength in the first 20-s (red box), fitted by a straight line. *n* = 11 neuron pairs are used and error bars indicate Mean ± s.e.m. **(C)** The simulated neural responses to 100-s flicking pseudo-random checker-boards. **(D)** The change of the correlation strength based on the short-term facilitation in Equation 6. Inset shows the change of correlation strength in the first 20-s (red box). **(E)** The change of electrical coupling strength due to the short-term facilitation during the adaptation. **(F)** The change of the correlation strength during the adaptation with a constant electrical coupling strength (*g*^es^ = 0.025 μS). Inset shows the change of correlation strength in the first 20-s (red box). The simulation is repeated 10 times and error bars indicate Mean ± s.e.m. The parameters τ^*f*^ = 10 s, τ^*l*^ = 50 ms, *u*^*f*^ = 50 μS, and other parameters are the same as in Figure 1.

As shown in Figure 4A, the responses of RGCs exhibits a clear adaptive behavior, in terms of that the firing rates of RGCs first increase quickly at the onset of the stimulation and then they decrease gradually to a much lower value. We measure during this adaptation process, how the correlation strength between neurons coupled by an electrical synapse changes with time. The result is presented in Figure 4B, which shows that the correlation strength first increases with time in the first 20 s (Figure 4B inset) and then decreases gradually.

The fact that the neuronal correlation increases whereas the firing rates attenuate at the initial stage of the adaptation is not a trivial property. According to the result in Figure 3, for fixed synapse strength, the neuronal correlation should decrease with the attenuation of firing rates. We therefore, suspect that an enhancement of the neuronal interaction efficacy is going on during the adaptation (see Discussion for alternative mechanisms). Furthermore, it has been shown that the plasticity of a chemical synapse is insufficient to induce large change in the correlation strength (Figure 2B). Thus, we propose that it is the short-term facilitation of the electrical synapse leading to this paradox phenomenon.

**Figure 5:**
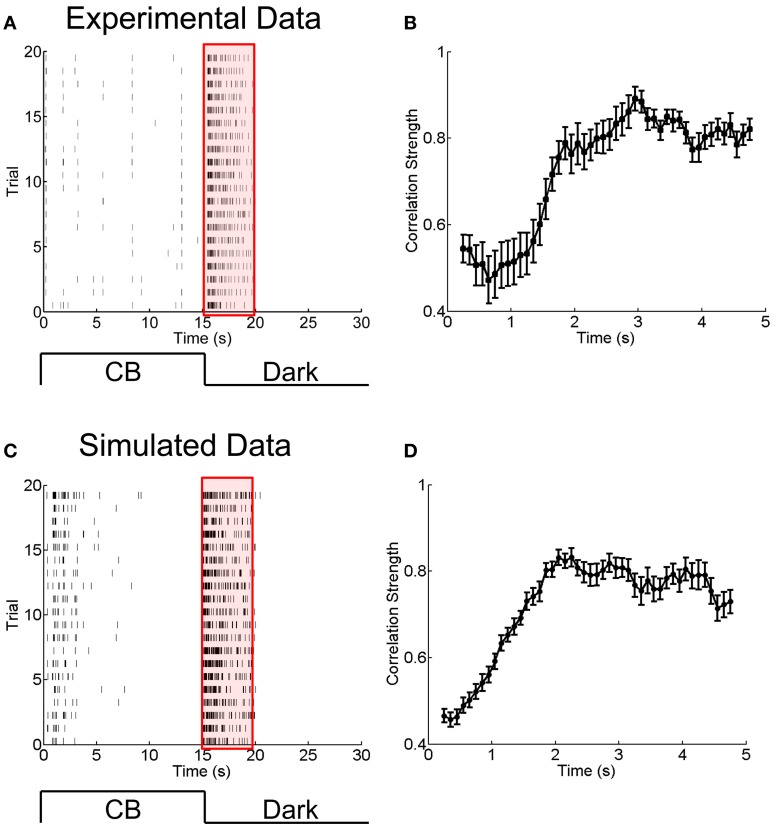
**Experimental and simulated results for the change of the correlation strength between two electrically coupled neurons during the adaptation to a sustained dark stimulation. (A)** An example of raster plots of a neuron's response to 15-s flicking pseudo-random checker-boards followed by 15-s darkness stimulation, repeated 20 times. Bin size is 2 ms. **(B)** The change of the correlation strength measured in the experiment during the adaptation to darkness (red box in A). *n* = 20 neuron pairs are used. Error bars Mean ± s.e.m. **(C)** and **(D)** Simulated results for the neuronal adaptive behavior and the change of the correlation strength when the short-term facilitation of electrical synapses is considered. The parameters are the same as in Figure 4.

To describe the experimental data, we propose the following phenomenological model for short-term facilitation of an electrical synapse, which is given by

(11) $$\tau^{f}\frac{dg^{\text{es}}}{dt}=-\left(g^{\text{es}}-g_{0}^{\text{es}}\right)+u^{f}\left(g_{max}^{\text{es}}-g^{\text{es}}\right)\exp\left(\frac{-\left|\Delta T\right|}{\tau^{l}}\right)$$

where τ^*f*^ is the time constant of short-term facilitation. *g*^es^_0_ and *g*^es^_max_ are the static and the maximum values of *g*^es^, respectively. Δ*T* denotes the time difference between two adjacent spikes generated by the two neurons. τ^*l*^ determines the time window for plasticity and *u*^*f*^ the rate of facilitation. This plasticity rule states that if two neurons fire strongly and synchronously in a short-time window, their electrical synapse is temporally enhanced. We fix the parameters in the model by the experimental data in Figures 4A,B. Once their values are determined, the model will be used to explain the results from another experimental data shown in Figure 5.

To mimic the luminance adaptation condition, we set the mean of the inputs to be μ(*t*) = 0.8*e*^−*t/a*^ with *t* = 0 being the moment of the stimulation onset. μ(*t*) decreases with time, reflecting that the current from bipolar/amacrine cells to a RGC attenuates during luminance adaptation (Baccus and Meister, 2002). We choose the parameter *a* = 20 s, so that the simulation results match the experimental data.

Combining Equations (1) and (11), we simulate the neuronal responses during the adaptation. Figure 4C displays how the firing rate of a neuron changes over time, which reproduces the adaption behavior observed in the experiment (Figure 4A). Figure 4D displays how the correlation strength changes over time, which reproduces the experimental observations shown in Figure 4B, namely, the correlation strength increases in the first 20 s and then decreases gradually to a stable value. This increment is due to the short-term facilitation of the electrical synapse in the first 20 s, as shown in Figure 4E. As a comparison, we also simulate the change of correlation strength between neurons when they are connected with constant electrical synapse strength (Figure 4F). In this case, the correlation strength linearly decreased with time during the adaptation process (the first 20 s; inset of Figure 4F), and is unable to explain the experimental observation.

### Computational role of dynamical electrical synapses

In the above we have demonstrated that short-term facilitation of electrical synapses can well justify the neuronal response properties during the adaptation. But, what is the functional meaning of this short-term plasticity?

To answer this question, we analyzed another set of experimental data in which the RGCs of a bullfrog retina were exposed to a sustained dark stimulation after having responded to flicking pseudo-random checker-boards for 15 s (see Materials and Methods). The whole adaptation process to the dark stimulus lasted for about 5 s. Figures 5A,B present the experimental results, which show that the firing rates of RGCs attenuated over time and that the neuronal correlation via electrical synapses increased over time. Similar to the analysis in section A Phenomenological Model for Short-Term Facilitation of Electrical Synapse by considering short-term facilitation of electrical synapses, our model, i.e., Equations (1 and 11), successfully reproduces the experimental data (Figures 5C,D).

To ascertain the computational contribution of the enhanced correlation and consequently the functional role of short-term facilitation of electrical synapses, we analyze how the stimulation information is encoded separately in the firing rates and the neural correlation during the adaptation. The information analysis approach is introduced in Materials and Methods.

**Figure 6:**
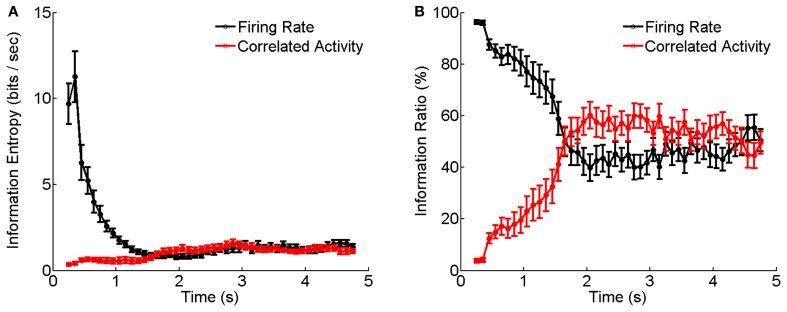
**Information entropy and information ratios carried by firing rate and correlated activity during the luminance adaptation. (A)** Information carried by firing rate and correlation of electrically coupled neurons during the adaptation to the dark stimulation. Bin size is 5 ms. **(B)** Information ratios carried by firing rate and correlation during the adaptation. *n* = 20 neuron pairs are used. Error bars Mean ± s.e.m.

Figure 6 shows the results calculated by Equations (6–10) based on the experimental data shown in Figure 5. We see that during the adaptation, the stimulus information contained in the firing rates decays dramatically with time, whereas, the stimulus information contained in the correlation of electrical coupled neurons tend to increase with time (Figure 6A). Their relative contributions exhibit a very interesting behavior: at the beginning of neuronal response to dark stimulation, more than 90% of the stimulus information is encoded by the firing rates; whereas after about 2 s, more than 50% of the stimulus information is encoded in the correlation (Figure 6B). This result implies that during the adaptation, there exists a transition in the encoding strategies of the neural system, namely, from the firing rate code to the correlation one, and a computational role of short-term facilitation of electrical synapse is to implement this transition operation.

## Discussions

In the present study we have investigated the potential computational roles of dynamical electrical synapses in neural information processing. We find that electrical synapses are more efficient than chemical synapses in modulating the concerted activity of neurons. That is because an electrical synapse tends to equate the sub-threshold membrane potentials of connected neurons and hence is more efficient in controlling synchronous firing of neurons. On the other hand, a chemical synapse only conveys signal when a neuron fires, and its effect in coordinating synchronous firing can be easily disturbed by fluctuations in sub-threshold potentials of the neurons due to input noises.

Based on the experimental data, we propose a phenomenological model for short-term facilitation of electrical synapses, which successfully reproduces the seemingly paradox phenomenon that the increment of neural correlation is associated with the attenuation of firing rates. In a recent work, Cortes et al. (2012). found that chemical synapses with neuronal spike-frequency adaptation can also generate this paradoxical behavior. Nevertheless, for the particular neural data considered in this study, namely, the responses of RGCs, we believe that short-term facilitation of electrical synapses is a more plausible mechanism for two reasons. First, RGCs are abundantly connected by electrical synapses, and their interaction through chemical synapses is indirect (mediated by bipolar and amacrine cells); and secondly, the *CCF* between RGCs measured in the experiment has a very narrow peak and it exhibits dual peaks when the bin size is sufficiently small, which are the typical syndromes of electrical synapses.

We investigated how the stimulus information is encoded separately in the firing rates and the correlations of RGCs during the luminance adaptation. We find that there exists a transition from the firing rate code to the correlation one at the late stage of the adaptation. The latter encodes the stimulus information by using the concerted, but less active, firings of neurons, and hence is economically more efficient. Our finding suggests that dynamical electrical synapses can play profound roles in neural information processing.

## Author contributions

Lei Xiao, Si Wu, and Pei-ji Liang designed experiments. Lei Xiao and Pei-ji Liang performed experiments; Lei Xiao, Dan-ke Zhang, Yuanqing Li, and Si Wu implemented simulations, and performed model analysis; and Lei Xiao, Si Wu, and Pei-ji Liang wrote the paper.

### Conflict of interest statement

The authors declare that the research was conducted in the absence of any commercial or financial relationships that could be construed as a potential conflict of interest.
